# Navigating uncharted territory: a qualitative study of the experience of transitioning to wheelchair use among older adults and their care providers

**DOI:** 10.1186/s12877-015-0092-2

**Published:** 2015-07-28

**Authors:** Edward M. Giesbrecht, William C. Miller, Roberta L. Woodgate

**Affiliations:** Department of Occupational Therapy, University of Manitoba, R106-771 McDermot Avenue, Winnipeg, Manitoba R3E 0T6 Canada; Department of Occupational Science and Occupational Therapy, University of British Columbia, T325-2211 Wesbrook Mall, Vancouver, BC V6T 2B5 Canada; Faculty of Nursing, University of Manitoba, 89 Curry Place, Winnipeg, MB R3T 2N2 Canada

**Keywords:** Wheelchair, Participation, Training, Social support, Confidence

## Abstract

**Background:**

An increasing number of older adults are procuring a wheelchair for mobility; however, the corresponding impact on related injuries, caregiver burden, and participation restriction is concerning. To inform the development of a wheelchair training program, we pursued a clearer understanding of the experience transitioning to wheelchair use for older adult users and their care provider.

**Methods:**

Six focus groups were conducted with older experienced wheelchair users (*n* = 10) and care providers (*n* = 4). Transcripts were analyzed using a Conventional Content approach; a coding framework enabled inductive theming and summary of the data.

**Results:**

Three themes emerged from the user group: *On My Own* reflected both limited training and the necessity of venturing out*, More Than Meets the Eye* addressing barriers to use, and *Interdependence* between wheelchair users and the ambulatory community. Care provider responses fell into two themes: the *All Encompassing* impact of assumed responsibilities and *Even the Best Laid Plans*, where unpredictable and inaccessible environments sabotaged participation.

**Conclusions:**

The transition from ambulatory to wheelchair mobility can feel like uncharted territory. Balanced support and appropriate mentorship are fundamentally important and real-world encounters optimize independence and proficiency with skills. The impact on care providers is extensive, highlighting the importance of skills training.

## Background

The wheelchair is becoming an increasingly common assistive device for older adults. With age, the risk of a disabling health condition increases and mobility is the most prevalent area of impairment among older adults in Canada [1]. A 2004 study reported that among multiple assistive device users, the manual wheelchair (MWC) was considered third most important, following eyeglasses and canes [2]. In fact, the wheelchair icon has become synonymous with accessibility. The number of wheelchairs provided to address mobility issues among older adults is rising. In 2001, an estimated 81,000 Canadians 65 years and older required a wheelchair for mobility [3]. The introduction of an assistive device is intended to improve mobility, function and quality of life as well as reduce the need for personal assistance and diminish burden for care providers [4]. However, the *acquisition* of a wheelchair does not necessarily mean the user will become independently mobile or improve performance of functional activities. In both Canada and the United States, over 90 % of older adult MWC users experience performance restrictions in at least one major life activity [1] compared with only 15 % for those who don’t use a mobility device [5]. To accomplish these activities, assistance must often be engaged from a family member or other care provider [6]. In Canada, nearly six in ten older adult wheelchair users require assistance from a care provider for basic mobility [3]. Compromised participation and social connectedness have been implicated with restrictions in mobility [7], often manifesting in experiences of isolation, stress and low self-esteem diminishing quality of life [8]. These challenges to independent mobility affect not only wheelchair users, but their families as well. A 2006 study of stroke survivors adjusting to wheelchair use identified substantial restriction in care providers’ social roles and an increased burden of care [9]. One quarter of all care providers for the elderly in Canada are themselves over 65 [10] and at increased risk for injury while assisting these wheelchair users.

Acquisition of a wheelchair, or any assistive device for that matter, is a complex and multi-step process, including comprehensive assessment, prescription, procurement, configuration, proper fitting, adequate training and follow-up [11]. Mortenson and Miller [12] have explored user and prescriber experiences of the wheelchair procurement process, and identified how physical, environmental and resource constraints often compromise the ability to secure optimal equipment and achieve desired goals. Beyond procurement, learning to effectively operate and maneuver the wheelchair in a variety of contexts is a critical factor. In fact, older adults’ acceptance of assistive devices, such as a wheelchair, depends greatly on the adequacy of training provided, particularly during initial acquisition [13]. While the evidence indicates structured training improves wheelchair mobility and operational skill, older adults typically receive little or no skills training. A recent survey of 68 Canadian rehabilitation centres reported only two-thirds offered basic skills training (e.g. propulsion), typically for 1–4 h, and advanced skills training (e.g. wheelies) was provided in less than 12 % of facilities [14]. This lack of training not only diminishes the user’s capacity to participate in meaningful activities, but also place them at greater risk for injury. The estimated annual incidence of tips and falls among Canadian wheelchair users is 5.2 %, with roughly 80 % incurring an injury and half resulting in an Emergency department visit [15]. Recent studies in the United States estimate the cost to treat such an injury is $25,000–75,000 and there is on average one fatality per week due to wheelchair-related accidents [16].

Despite the documented rise in use, little is known about the user experience of transitioning from ambulatory to wheelchair mobility, particularly among older adults; supporting such mobility transitions has been an identified need [8]. Furthermore, the interplay between the wheelchair user and their care provider (who is often a spouse or family member) during this process is poorly understood. To inform the development of a novel wheelchair skills training program specifically for older adults [17], we pursued a clearer understanding of the lived experience of this transition to wheelchair use, particularly the challenges and facilitators encountered by participants. Our research question examines “what is the process and impact of adjusting to wheelchair use for both older adult users and their care providers?”

## Methods

As part of a larger training program development study [18] employing a Participatory Action Design approach [19], six focus groups were conducted across two large metropolitan centres in Canada (Winnipeg and Vancouver). We used focus groups as a qualitative research strategy because the experience of transitioning to wheelchair use is a relatively unexplored phenomenon and difficult to ascertain through quantitative means. A focus group format provided an organic means to elicit participants’ experiences and uncover underlying contributing factors, rather than seeking confirmation for *a priori* assumptions. Rather than individual interviews, focus groups were intentionally used to promote exchange and dialogue, drawing out less vocal participants, and fostering both common and diverse experiences. Participants were recruited through public advertisement and direct invitation via consumer advocacy agencies, rehabilitation hospitals, and research lab databases. A total of four focus groups were undertaken with older experienced MWC users (*n* = 10), consisting of two sessions, conducted 3 months apart in each city. In Vancouver there were six participants and in Winnipeg there were four. Two focus groups (*n* = 4) were also conducted with care providers (i.e. a spouse, family member, or paid caregiver of an older MWC user), one in each city. We had targeted 3–6 participants for each group to ensure a balance between breadth of experience and opportunity for participant engagement, and to be able to pragmatically gather on multiple occasions [20]. Recruitment of care providers proved to be considerably more challenging due to limited avenues of access, poor response to advertisement, and the fact that many older MWC users did not have an interested care provider. Written informed consent was obtained from all participants prior to conducting the focus groups and study approval was obtained from the Research Ethics Boards at the University of Manitoba (#H2011:357) and the University of British Columbia (#H11-02558).

One of the authors (EMG), with previous facilitation experience and expertise in the content area [21], facilitated all focus groups together with a Research Assistant (RA). Each focus group was approximately 2 h in length and included an introduction identifying the purpose of the study, agenda for the session process, and procedures for analyzing and sharing data. Discussion was initiated using a semi-structured guide with broad questions informed by our review of the literature; prepared follow-up questions and probes to elicit additional information; and spontaneous questions responsive to content raised by participants. In particular, questions related to the experience of transitioning to wheelchair use (e.g. “Tell me about your experience using a wheelchair/providing assistance?”); developing proficiency with use and the impact on function (e.g. “Tell me about how you learned to use your wheelchair/what skills have been most useful”), and barriers to use (e.g. “Tell me about situations or activities that have been most challenging”).

For the care providers, the facilitation process was comparable but the discussion guide focused primarily on recalling how the transition to wheelchair use had impacted their life individually as well as collectively with the wheelchair user. Questions related to demands encountered (e.g. “What has been your experience with their use of a wheelchair; do they require your assistance”) and the implications for their lived experience (e.g. “Does their (in) ability to operate the wheelchair affect activities they/you choose to engage in?”). The first author kept field notes related to session content and personal interpretations while the RA kept field notes on session process and participant interaction. All sessions were audio-recorded and video-recorded to capture non-verbal communication for later review. Audio-recordings were transcribed verbatim by the facilitating RA and a second RA verified transcription accuracy against the audio recordings before removing personal identifiers.

### Data analysis

Our intention was to explore the phenomenon of participants’ lived experience. Given the absence of any predisposing theory due to limited research in this field, we analyzed the transcripts using a Conventional Content approach [22], allowing insights to emerge inductively from the text. The first author reviewed each transcript multiple times to become immersed in the data. Content from the first transcript was parsed into elements capturing discrete thoughts or concepts, with codes formulated for each. This process was repeated with each subsequent transcript, integrating existing and emergent codes. After completing this initial open coding, the data was reconstructed and reduced into complementary axial codes reflecting broader conceptual issues; documented inclusion parameters were created to delineate concepts. Using an iterative and reflexive process, data were then consolidated into overarching themes unique to the MWC user and care provider groups. Team members proposed themes that explicated congruity and conveyed underlying commonalities between multiple participant experiences. The MWC user themes were reflective of participants’ experience accepting changes in their mobility and how they adapted accordingly. Care provider themes revealed the impact of this transition on their personal and shared experiences. Analyses of MWC user and care provider groups were conducted in parallel to provide contextual insight, but independently coded and themed. The literature suggests that the experiences of user and care provider are sufficiently different [9] and our intent was to explore these unique experiences in depth rather than conflate them. Furthermore, the contrasting foci (i.e. adapting to wheelchair use versus adapting to living with a wheelchair user) created complementary rather than collective insights.

The research team communicated regularly and all authors reviewed coding; any discrepancies were discussed until consensus was reached. An audit trail of the research and analysis process, including all coding procedures, was documented. To enhance credibility of the data, the first author had extended engagement and communication with participants throughout the broader program development study and engaged them in member checking following the focus group analyses. To ensure anonymity, participants’ names were replaced with pseudonyms in the transcriptions used for analysis and in this report.

### Participants

#### MWC users

Ten individuals agreed to participate; six in Vancouver and four in Winnipeg, with only one female at each site (Table 1). Participants were required to be 55 years of age or older, live independently in the community, use a MWC as their primary means of mobility for at least 1 year and have sufficient cognition and English language skills to engage in a focus group. Some participants had made their transition to wheelchair use later in life while others were more experienced, having acquired their wheelchair in early- to mid-life, and all were dealing with the effects of aging on wheelchair use. All of these participants, with the exception of Mike and Brent, were active and independent users spending at least 8 h a day in their wheelchair engaging in both indoor and outdoor activities. Mike and Brent were independent with indoor mobility in accessible environments, but required assistance when encountering outdoor or challenging indoor situations and when propelling long distances.

**Table 1:** Description of wheelchair user focus group participants

Pseudonym	Age range	MWC experience	Formal skills training	Site
Tim	65–74	50 years	Yes	Vancouver
Mike	75–84	4 years	No	Vancouver
Louise	55–64	48 years	No	Vancouver
Vern	55–64	39 years	Yes	Vancouver
Ted	55–64	11 years	Yes	Vancouver
Richard	55–64	23 years	No	Vancouver
Michelle	55–64	60 years	No	Winnipeg
Frank	65–74	25 years	No	Winnipeg
Brent	75–84	37 years	No	Winnipeg
Allen	75–84	15 years	No	Winnipeg

#### Care providers

Participants were individuals who provided assistance to or accompanied a MWC user over 55 years of age inside or outside of the home on a regular basis. Two females in each city agreed to participate (*N* = 4). In Winnipeg, Jamie worked as a care provider in a communal living complex that included individuals with a disability (including older adult MWC users) and Felicia provided care for her husband who was in his 70s and a long-time wheelchair user. In Vancouver, Bertha provided care for her adult daughter and Patricia for her husband.

## Results

### The wheelchair user experience

**Fig. 1:**
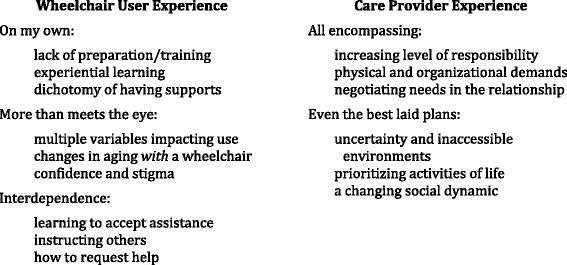
Overview of themes and subthemes

Participants in the two user groups identified a variety of issues that had impacted their transition to wheelchair use, which are summarized in three overarching themes (Fig. 1).

#### Theme 1: on my own

Seven of the ten participants indicated they had received little preparation for the demands of navigating their wheelchair in an “ambulatory world”. Three participants shared similar experiences receiving some degree of wheelchair mobility skills training (having all attended the same rehabilitation centre), such as Tim who said: *“I came through [Rehabilitation Facility] and learned quite a bit from the staff but found that getting out at home and the park and other places, encountered things that I didn’t experience [in Rehab]”.* However, most participants reported little or no specific skills training to prepare them for navigating barriers in the community, even those who had received formal instruction in hospital. Michelle said she learned *“by trial and error, I don’t remember any formal or informal contact with any professional”.* Frank reflected on how *“I realized I have to do this now because … it’s with blood and tears all the time, you know, we own this … a lot of getting used to different things.”* Mike highlighted the frustration of not having access to potential resources: *“I know there is a lot of training and knowledge out there but it seems it isn’t handed out, there was no system of giving [training]”.*

Learning to use a wheelchair was perceived as an experiential undertaking–in order to learn how to navigate the world you must explore your environment, as Frank puts it: *“Hey, I had to do it [then] because look, somebody’s … not going to be there all the time, so we need to know [how to do it]”.* Participants spoke often about the need for exposure to new situations so they could master wheelchair maneuvers and generalize the skills they had acquired to novel and increasingly challenging environments:

*“[the most effective way to learn skills is] going out and doing them … the first time I do something I usually try to take somebody who … can help me get through it if I really have difficulty … and then once I know that I can cope, then I can do it on my own forever after”.* [Louise]

Other experienced wheelchair users, particularly contemporaries, were a source of some benefit. In particular, the three participants who had received formal training during their rehabilitation stay identified peers within the facility that provided some form of instruction and motivation. Participants recalled that learning sometimes transpired through intentional means. For example, Vern identified: “*peers and mentors that would show you tricks that they learned over the years having used a wheelchair*”. However, this wasn’t always engendered through a direct relationship and in some cases mentoring was more subtle and detached:

*“If you see some active people doing things and start to think about what it is they’re doing differently … because they’re not going to explain it to you, it’s something that you learn by … seeing them doing it”.* [Tim]

An interesting conundrum emerged during the participants’ exchange; support from significant others was identified as both a help and hindrance to wheelchair mastery. Michelle stated she never really learned wheelchair independence until she moved out after university:

*“[At home] if I needed to go up a ramp, somebody was there to push me … it wasn’t probably until I was in my twenties and had my own apartment and then had a car that I then started doing things on my own”.*

Another example of this dichotomy, for those who acquired a wheelchair later in life, revolved around having a partner. Frank related his experience 25 years earlier as a single man learning to adapt to mobility with a wheelchair, where practicing was particularly difficult and fraught with risk: *“I was quite prone to accidents because I did some things just where I had to learn, and I learned the hard way because when I got out of the hospital, my wife took off so I was on my own”.*

#### Theme 2: more than meets the eye

Participants identified that effective wheelchair use resulted from a multifactorial interplay between the individual, the activities they pursue, and environmental variables. Environmental factors related to accessibility, such as ramps and curb cuts, were essential to have the *opportunity* to practice mobility skills, as well as the wheelchair device itself. The type of wheelchair obtained, such as a lightweight rigid-frame chair that required less effort to push and allowed for greater customization, impacted participants’ capacity for active mobility:

*“Choosing the right chair for the right person is very, very important. I hate those old clunkers that they give to people and they can’t do anything with them, they’re so heavy and so awful and they can hardly wheel them”.* [Allen]

Participants further stipulated that proper *configuration* of the wheelchair could enhance performance. Adjustments by the clinician, such as position of the rear drive wheels, could influence ease of propulsion, maneuverability, and ‘tippiness’. Frank related his experience returning to work after acquiring his wheelchair: *“I had so many falls because … I’d go and pick something up from the floor and I fell over because the wheels weren’t set properly”.*

Intrapersonal attributes, such as level of impairment and strength, were perceived as relevant to the process of adapting to wheelchair use and impacted the kinds of mobility skills that were reasonable to acquire. Age-related changes further affected performance and required *continual* adjustment to wheelchair use:

*“Complications that came about [from] aging in a wheelchair - the shoulders give out, the wrists give out, and so it’s trying to adapt to those things … now I have to almost do it a different way again in order to maintain that level of independence you’d like”.* [Ted]

Brent noted that some skills acquired earlier in life were now used reluctantly or not at all because older wheelchair users were less physically able and more risk-averse: *“I can’t bend over as much as I used to, I can’t jump curbs, going up ramps is a little bit difficult ‘cause I can’t lean forward as much as I used to, to get my balance”.* Allen articulates his growing concerns about higher-risk activities as he gets older:

*“I’m afraid of a wheelie … even though I’m [an experienced user] because I’m always thinking ‘am I going to get wheeled too far and go right over?’.”*

The issue of confidence in one’s capacity to learn and perform more advanced wheelchair maneuvers was salient. Louise observed “*You get into situations where there’s generally a solution but if you haven’t had any experience then you’re hesitant, right?”* Self-confidence was linked to internal and external stereotypes of aging. Participants noted that they had their own preconceptions about lacking the capacity to acquire and master advanced skills–these were things that young people did but would be too difficult for older adults. Allen articulates this position, that *“you’ve got some people who say ‘I’m in a wheelchair, I’m old, I can’t do anything, I need somebody else along to push me, I can’t do it’”.*

Confidence and persistence in learning to use the wheelchair were also linked to the psychological and affective predisposition of the individual. Participants related that *acceptance* of the wheelchair was a primary key to improving mobility, and the loss associated with compromised ambulation was closely tied to investment and motivation in learning mobility skills. Frank recalls this emotional transition:

*“My friends said ‘look at him in a wheelchair’ and laughed at me, you know? I didn’t realize, my god this is a way of life I have to live for the rest of my life … I got myself started this way, and I eventually started to feel comfortable in my own skin*”.

Such notions were borne not solely from self-image, but also broader cultural perceptions of what older adults are capable of:

*“Because [we’ve] had all these preconceived notions about wheelchairs and what you can and can’t do … by being in the world or seeing things or watching TV … [we’re] going to think ‘oh, I’m never, I’m not doing that’.”* [Michelle]

#### Theme 3: interdependence

A third emergent theme was the sense of interdependence between those who use a wheelchair and those who don’t. Participants reflected on the importance and necessity of *collaborating* with non-users. Several subtle variants on this theme came to light: learning to accept and use assistance; knowing how to instruct the novice helper; and knowing how to ask for help. As part and parcel of embracing the transition to wheelchair use, learning *when* to seek assistance from others was pertinent. Allen recognized situations where the risk of injury was unreasonable, such as icy streets in winter where his wheelchair was prone to slide during transfers into the car, so he chose to stop a passer-by to aid in stabilizing his chair. Participants also identified situations where they might be capable of independent mobility but it was simply easier, safer, or more expeditious to ask for (or accept) assistance. This was perceived as being selective about when and where to expend effort, rather than inability: *“I never refuse somebody who is going to push me up a ramp. Why, if somebody is there to push me up a ramp, should I be working - I mean, I can do it, but so what?”* [Allen]

There was overwhelming agreement that it was equally important to learn how to instruct others in providing safe and effective assistance. Taking control and being directive with the helper was identified as critical, particularly in situations where the risk was elevated:

*“I’ll ask people for help myself and they’ll approach the [ascending] stairs forwards and I’m thinking ‘don’t do it that way’, so I say ‘no, you’ve got to turn me around, one person here, one person here’.”* [Michelle]

Finally, participants spoke about learning *how* to request assistance from others, and advocating courtesy when assistance is offered, even when it was not required or desired. Allen offers this advice:

*“Wheelchair manners [are important] too - if you don’t want [help] to do it, thank the person, accept gracefully and appreciate them … a responsibility to be courteous as a wheelchair person … because they’ll go offer somebody else”.*

Furthermore, Michelle speculated that a negative encounter might have future repercussions:

*“Invariably there are people out there who have offered help to somebody who is disabled and had their head chewed off for it so the next disabled person they see, they’re very reluctant – they’re just ‘Oh I don’t need that again, I’m going to walk on by’.”*

### The care provider experience

Care providers also identified a variety of issues and experiences that accompanied transition to wheelchair use by the older adults with whom they were closely involved, as illustrated through two broad themes (Fig. 1).

#### Theme 1: all encompassing

Care providers reflected on how their level of responsibility increased dramatically with the transition to wheelchair use. The demands that now fell their way were substantial, particularly in how responsibilities felt unrelenting and pervasively entered so many areas of life. Some of these responsibilities were foreign to the care providers, but assuming them was the only viable option. Patricia recalls the demands incurred when her husband transitioned to using a wheelchair: “*It’s all very practical, there’s repairing the chair – if he gets a flat tire … those little things – they don’t happen everyday, but when they happen you’re usually the one who’s around”.*

Most apparent were the physical demands of providing assistance such as assisting the user to transfer in and out of their wheelchair; pushing or maneuvering the wheelchair in difficult or less-accessible situations; or driving to a destination and having to dismantle and lift the wheelchair into a vehicle. Bertha, already into her 80s, spoke about helping her daughter: “*You have to have strength enough to – [help] if they get into difficulties … [she] has tipped out of her chair several times over the years and you have to be able to get her back into the chair again.”* There was concern about the significant risk with assisting and how this hazard was exacerbated with aging–their own physical ability to perform tasks and the user’s diminishing capacity:

*“If they fall out, there’s some problems … as he’s gotten older – needing help with transfers … I think part of it is the physical strength, but also … figuring out how he can best help me and then me not having to hurt myself”.* [Patricia]

Despite these physical demands, care providers identified greater concern about the other requirements placed on them, such as the need to schedule, organize and manage the MWC user’s life. Even when the user was capable and largely independent, care providers felt that ultimately *they* assumed responsibility for organizing appointments and transportation matters or anticipating issues that might impact participation in activities outside the home. Patricia spoke about situations where shops and businesses were inaccessible, such as a step or series of steps to get into a fast food restaurant. In addition to purchasing the food, she also felt compelled to make all the decisions:

*“You go to a deli – ‘what kind of sandwich would you like?’ – because he can’t get in the door – so then you gotta remember the type of sandwich … it’s like ‘with fries, without fries’ – you have to kind of guess what [he] would like”.*

Frequently, negotiation between the care provider’s own social activities and those of the MWC user required compromise. Care providers who were a spouse or parent of the user talked about how these responsibilities were ‘part of the package’ of their relationship. However, Felicia also revealed that, at certain times in her life, she wondered about the fairness of her circumstances:

*“[For] 5 years … the whole situation depended on me to be there every second for every movement … I don’t think it was time [commitment], it was more of the ‘hey did I sign up for this?’ … We’ve been married for 48 years - was that what they meant in the vows? Was I going to do this forever?”*

#### Theme 2: even the best laid plans

Uncertainty and pseudo-accessible environments [23] exacerbated this sense of all-encompassing responsibility; despite scheduling and organizing activities, these plans were invariably subject to revision and sabotage. Patricia reflects an example:

*“No matter how much you phone ahead and [ask] ‘are there any stairs?’ – ‘No problem, you can get in’ – so we get there and there’s two stairs into the front door … it’s just that feeling of – we even phoned ahead and it’s still a problem”.*

Jamie described a situation where several wheelchair users for whom she provides care were offered complimentary tickets to a theatre show but, upon arrival, were unable to access the ‘accessible’ seating location. Several participants spoke of having to ‘expect the unexpected’ and the unpredictability of community environments. In addition to having to make multiple decisions, care providers were also required to make them ‘on the fly’ in a dynamic and time-sensitive context.

*“[My husband] wheeled over some thorns in a park – ‘pop’ – the tire blows up. So then it’s trying to figure out where is there a medical supply place [or] bicycle store –and you’re trying to remember all the details”.* [Patricia]

These unpredictable scenarios often occurred in a crowded public location, creating anxiety for both wheelchair user and care provider. Jamie talked about how this meant she was responsible for not only her own anxiety, but also that of the wheelchair user, and the tremendous social pressure to resolve issues expeditiously, which then increased the risk of injury from assisting too quickly or ‘cutting corners’.

These complications compelled care providers to prioritize some activities at the expense of others, and there were invariably missed opportunities for social engagement. Felicia spoke about their experience during her husband’s transition to wheelchair use: *“We tended to focus on things you had to do … and learned how not to do a lot of things*”. Participants spoke of a narrowing of social circles for both the user and the care provider. Felicia reminisced about her husband’s numerous leisure interests and social groups, and how these opportunities began to vanish because meeting locations were insufficiently accessible. Diminishing opportunities for socialization also applied to mutual activities where socially invoked expectations of participating ‘as a couple’ precluded an invitation.

Even social gatherings with family and friends were influenced by the transition to wheelchair use. Bertha identified the homes of her children had stairs at the front door and were not wheelchair accessible. Patricia noted both she and her husband were becoming more risk-averse and were not willing to have him be ‘carried up the stairs in his wheelchair’ when visiting family: “*we don’t go there anymore because we’re all getting older - he doesn’t want to fall out of his chair*”. As a result, family gatherings and rituals, such as Thanksgiving dinner, were no longer held in a family member’s home but an accessible restaurant instead. While they continued to share these collective events, the dynamic was clearly different and there was a sense of loss that came with celebrating intimate events in a very public venue.

## Discussion

Despite the relatively high prevalence of wheelchair use among individuals with mobility impairment, participants described the journey of transition as lacking any sort of roadmap or guidance. Many identified feeling isolated and ill-prepared to adapt to changes that necessarily occurred when the wheelchair became a ubiquitous consideration of daily life. The challenge of learning how to operate the wheelchair in a variety of environments and conquer accessibility obstacles was daunting and often discouraged efforts to participate in prior activities. The narrowing of social circles and discriminatory conventions of social engagement often exacerbated this experience. Some wheelchair users identified positive experiences during the initial period of transition, through a supportive community of therapists, peers and experienced wheelchair users within a rehabilitation facility. However, most never had access to such a venue and these disenfranchised wheelchair users were essentially left to their own devices, learning principally through trial and error.

Regardless of whether they received any preparation as novice users, participants universally agreed learning to use their wheelchair demanded they venture into the community. This could be a very difficult choice, given the risks associated and their lack of confidence. However, there was a strong sense that independent mobility was a direct consequence of choosing to conquer real-world obstacles. This involved not only learning skills, but also learning to adapt those skills and problem-solve dilemmas that arose because of the varied nature of environments encountered. The concept of generalizing skills through contextual learning is well supported in the motor learning literature. Studies have demonstrated training that incorporates contextual interference [24], or variations in skill and situation, produces better retention and improved skill performance in novel situations [25]. These findings suggest that a wheelchair training program delivered in a community context, such as a home program, has a strong potential for integration of mobility skills.

That the undertaking of community mobility was tied to personal supports and social resources presented somewhat of a conundrum, since these could operate as both facilitators and barriers to independence. On the one hand, individuals with strong familial supports, like Michelle, needed to break free in order to acquire the necessary skills. Conversely, those without a support system were necessarily ‘thrust into the fire’ and compelled to learn how to manage their wheelchair independently at some considerable risk. While the potential for injury or becoming stranded was high and created considerable anxiety, the impetus to gain mastery and independence could also be a strong motivator, as in the case of Frank.

Participants identified that, rather than any one single factor, multiple variables contributed to optimizing wheelchair use. Attributes of the individual, such as physical ability, self-image and confidence, impacted their capacity to master wheelchair mobility. Recent studies lend support to the relationship between self-efficacy, wheelchair proficiency and community participation [26]. Development of a wheelchair training program should incorporate principles of self-efficacy theory to enhance learning and acquisition of mobility skills. Participants noted the environment was equally influential in successful wheelchair use. Accessibility of the physical environment; support and acceptance in the social environment; and appropriate selection and configuration of the wheelchair device itself were variables of impact. Finally, participants made influential decisions around engaging (or not) in varying types of activities and occupations, particularly those previously enjoyed. The interplay between these factors–the person, their environment (including the wheelchair device), and the activities they choose to engage in–is synchronous with theoretical models in rehabilitation and research related to mobility among older adults [8]. For example, conceptual frameworks in occupational therapy (e.g. Canadian Model of Occupational Performance) [27] and assistive technology outcomes (e.g. Human Activity Assistive Technology model) [28] situate functional performance as a consequence of the fit between these components. When one component is suboptimal, overall participation can be compromised, despite adequacy in the remaining elements.

Participants made a particular link between the need for support and the social environment. Beyond their immediate network of care providers, they inevitably encountered situations where assistance from strangers was required. These wheelchair users perceived a broader social relationship, where societal interdependence had a mutually beneficial outcome for both the helper and the one being helped. This relationship was cultivated during encounters where wheelchair users asked for or were offered assistance. An intriguing notion raised was the role of courtesy, extending even to the point of civic responsibility. Underlying this perspective, these encounters create an experience or memory that impacted the likelihood the ‘other’ would provide assistance again in the future. A positive perception would leave a lasting impression enticing the helper to offer assistance to others in the future, essentially ‘paying it forwards’. Conversely, a negative encounter was thought to poison the well of future opportunities. In short, these *individual* encounters were thought to reflect positively or negatively on wheelchair users *collectively*.

Whether this perception is indicative of wheelchair users generally, or older adult users specifically, or a generational bias from a time when civic responsibility and civility was more explicitly engendered, is not clear. However, participants felt it was a step towards enhancing *reciprocity* between the ambulatory and wheeled-mobility worlds; that asking, receiving and providing assistance serve to promote collaboration and could, in some sense, offer a mutually-beneficial experience, as Allen notes: *“People are delighted to be able to help someone, it makes them feel good, it makes me feel good”.* Social exchange theory suggests that the mode of exchange between individuals can influence future behaviour, and that rewarded action, such as offering assistance, is more likely to be repeated [29]. Inherent in social exchange theory is the concept of interdependence, where individual human interactions are linked to the broader social structure and a sense of reciprocity from the mutual benefit of these actions [30]. It has been reported that elders with declining function find it emotionally difficult to ask for or receive assistance [31]. Those who experience a positive relational exchange with their helper tend to be more accepting of assistance because of the perceived reciprocity in the encounter, even if the benefit to the helper is simple gratitude [32].

Despite the belief that wheelchair acquisition reduces caregiver burden, care providers in this study reported demands as widespread and overwhelming. The expectations extend beyond simply the physical demands of pushing and transferring the user, but also assuming the roles and responsibilities the user previously performed, including the mundane. Perhaps more encompassing were the planning and decision-making responsibilities. Care providers lamented the loss of spontaneity and the effort required to arrange an outing, whether it was for them alone, the wheelchair user, or both collectively. Prioritizing and choosing activities, particularly which ones to dismiss, could be an onerous task and, for some, challenged their perception of equity in the relationship. There were numerous physical and social barriers that further sabotaged plans, such as encountering a venue that lacked accessibility as promised. Both care providers and MWC users commented on the issue of inaccessibility, as it had a mutual impact. In such situations, the MWC user invariably relied on their care provider for assistance and there was often a need for a collaborative resolution. These findings suggest that wheelchair training should ideally involve the MWC user and any care providers. Providing instruction on multiple approaches or methods to address environmental barriers (i.e. independent, assisted and collaborative) could alleviate some care provider demands and increase the repertoire of strategies available in novel situations.

In light of the experiences reported here, further study should be undertaken exploring the extent and scope of training provided for older adults transitioning to wheelchair use, and the impact of such training, or lack thereof, on social participation. In particular, the efficacy of education and training strategies specifically tailored to older adults should be investigated, such as the use of peer-trainers and mentors. While this study presents some novel and revealing insights in the experience of older adult wheelchair users and their care providers, some limitations should be noted. The two study sites were quite diverse in many respects (e.g. geography, climate, racial diversity and wheelchair accessibility), but experiences in other cities or rural locations might be quite different. While we did not collect socio-economic data from the participants, they were typically middle-class and mobile; many wheelchair users are financially disadvantaged with limited means of transportation and a more diverse participant group might uncover other experiences. Despite substantive recruitment efforts, the number of care providers in this study was smaller than desired and this may have restricted the breadth of responses. All of the care providers in this study were female and most were a spouse or parent; the experience of male care providers, children of MWC users, and those without a direct familial association might be very different. Finally, the participants were a composite of elders who had experienced the *transition to MWC use* and those experiencing the *transition of aging as a MWC user*; conflating these experiences might have diluted the interpretation and findings of the study. However, obtaining this breadth of experience and explicating commonalities of older adults aging with and transitioning to wheelchair use was informative, including the need to re-learn many aspects of wheelchair use as a result of changing capacity with age. Caution should be exercised in generalizing findings to individuals who are substantially dissimilar to the participants and their situations as described herein.

## Conclusions

The transition from ambulatory to wheelchair mobility can feel like uncharted territory for older adults and their care providers, as only a select few receive training and mentorship. While support is fundamentally important, wheelchair users need to experience real-world encounters to optimize their independence and proficiency with wheelchair mobility skills. The impact of this segue into wheelchair use has a profound impact on care providers, particularly when they are a spouse or family member. These findings suggest that training is a critical component in wheelchair provision and informed development of our program using a community-based approach, integrating self-efficacy principles, and targeting both user and care provider.

## Abbreviations

MWC: Manual wheelchair
RA: Research assistant

## References

[1] Statistics Canada (2008). Participation and activity limitation survey 2006: a profile of assistive technology for people with disabilities.

[2] Mann WC, Llanes C, Justiss MD, Tomita M (2004). Frail older adults’ self-report of their most important assistive device. Occup Ther J Res.

[3] Shields M (2004). Use of wheelchairs and other mobility support devices. Health Rep.

[4] Chen T, Mann WC, Tomita M, Nochajski S (2000). Caregiver involvement in the use of assistive devices by frail older persons. Occup Ther J Res.

[5] Kaye H, Kang T, LaPlante HG (2000). Mobility device use in the United States.

[6] Hoenig H, Taylor DH, Sloan FA (2003). Does assistive technology substitute for personal assistance among the disabled elderly?. Am J Public Health.

[7] Finlayson M, van Denend T (2003). Experiencing the loss of mobility: perspectives of older adults with MS. Disabil Rehabil.

[8] Turner Goins R, Jones J, Schure M, Rosenberg DE, Phelan EA, Dodson S, Jones DL. Older adults’ perceptions of mobility: a metasynthesis of qualitative studies. The Gerontologist 2014, Advance access published March 17, 2014, 1–15. doi:10.1093/geront/gnu014. 10.1093/geront/gnu014 24637252

[9] Laliberte-Rudman D, Hebert D, Reid D (2006). Living in a restricted occupational world: the occupational experiences of stroke survivors who are wheelchair users and their caregivers. Can J Occup Ther.

[10] Cranswick K, Dosman D (2008). Eldercare: what we know today.

[11] World Health Organization (2008). Guidelines on the provision of manual wheelchairs in less resourced settings.

[12] Mortenson WB, Miller WC (2008). The wheelchair procurement porcess: perspectives of clients and prescribers. Can J Occup Ther.

[13] Kraskowsky LH, Finlayson M (2001). Factors affecting older adults’ use of adaptive equipment: review of the literature. Am J Occup Ther.

[14] Best KL, Routhier F, Miller WC (2015). A description of manual wheelchair skills training: current practices in Canadian rehabilitation centers. Disabil Rehabil Assist Technol.

[15] Kirby RL, Ackroyd-Stolarz SA, Brown MG, Kirkland SA, MacLeod DA (1994). Wheelchair-related accidents caused by tips and falls among noninstitutionalized users of manually propelled wheelchairs in Nova Scotia. Am J Phys Med Rehabil.

[16] Gavin-Dreschnack D, Nelson A, Fitzgerald S, Harrow J, Sanchez-Anguiano A, Ahmed S (2005). Wheelchair-related falls: current evidence and directions for improved quality care. J Nurs Care Qual.

[17] Giesbrecht EM, Miller WC, Eng JJ, Mitchell IM, Woodgate RL, Goldsmith CH (2013). Feasibilitiy of the Enhancing Participation In the Community by improving Wheelchair Skills (EPIC Wheels) program: study protocol for a randomized controlled trial. Trials.

[18] Giesbrecht EM, Miller WC, Mitchell IM, Woodgate RL (2014). Development of a wheelchair skills home program for older adults using a participatory action design approach. BioMed Res Int.

[19] Whyte WF, Greenwood DJ, Lazes P (1989). Participatory action research: through practice to science in social research. Am Behav Sci.

[20] McLafferty I (2004). Focus group interviews as a data collection strategy. J Adv Nurs.

[21] Giesbrecht EM, Ripat JD, Cooper JE, Quanbury AO (2011). Experiences with using a pushrim-activated power-assisted wheelchair for community-based occupations: a qualitative exploration. Can J Occup Ther.

[22] Hsieh H, Shannon S (2004). Three approaches to qualitative content analysis. Qual Health Res.

[23] Ripat J, Woodgate R (2012). Self-perceived participation among adults with spinal cord injury: a grounded theory study. Spinal Cord.

[24] Shea JB, Morgan RL (1979). Contextual interference effects on the acquisition, retention, and transfer of a motor skill. J Exp Psychol.

[25] Hanlon RE (1996). Motor learning following unilateral stroke. Arch Phys Med Rehabil.

[26] Sakakibara BM, Miller WC, Eng JJ, Backman CL, Routhier F (2013). Preliminary examination of the relation between participation and confidence in older manual wheelchair users. Arch Phys Med Rehabil.

[27] Townsend EA, Polatajko HJ (2007). Enabling Occupation II: Advancing an occupational therapy vision for health, well-being, and justice through occupation.

[28] Cook A, Miller-Polgar J (2008). Cook & Hussey’s assistive technologies: principles and practice.

[29] Emerson RM (1976). Social exchange theory. Annu Rev Sociol.

[30] Lawler EJ, Thye SR (1999). Bringing emotions into social exchange theory. Annu Rev Sociol.

[31] Allen RES, Wiles JL (2013). Receiving support when older: what makes it OK?. Gerontologist.

[32] Lewinter M (2003). Reciprocities in caregiving relationships in Danish elder care. J Aging Stud.

